# Effect of maternal age on the risk of preterm birth: A large cohort study

**DOI:** 10.1371/journal.pone.0191002

**Published:** 2018-01-31

**Authors:** Florent Fuchs, Barbara Monet, Thierry Ducruet, Nils Chaillet, Francois Audibert

**Affiliations:** 1 Division of Obstetric Medicine, Department of Obstetrics and Gynecology CHU Sainte Justine, Montréal, Québec, Canada; 2 Inserm, CESP Centre for research in Epidemiology and Population Health, U1018, Reproduction and child development, Villejuif, France; 3 Department of Obstetrics and Gynecology CHU Montpellier, 371 Avenue du Doyen Gaston Giraud, Montpellier, France; 4 CHU Sainte-Justine Research Center, Université de Montréal, Montréal, Québec, Canada; 5 Clinical Research Center Étienne-Le Bel, CHU Sherbrooke, Sherbrooke, Québec, Canada; Centers for Disease Control and Prevention, UNITED STATES

## Abstract

**Background:**

Maternal age at pregnancy is increasing worldwide as well as preterm birth. However, the association between prematurity and advanced maternal age remains controversial.

**Objective:**

To evaluate the impact of maternal age on the occurrence of preterm birth after controlling for multiple known confounders in a large birth cohort.

**Study design:**

Retrospective cohort study using data from the QUARISMA study, a large Canadian randomized controlled trial, which collected data from 184,000 births in 32 hospitals. Inclusion criteria were maternal age over 20 years. Exclusion criteria were multiple pregnancy, fetal malformation and intra-uterine fetal death. Five maternal age categories were defined and compared for maternal characteristics, gestational and obstetric complications, and risk factors for prematurity. Risk factors for preterm birth <37 weeks, either spontaneous or iatrogenic, were evaluated for different age groups using multivariate logistic regression.

**Results:**

165,282 births were included in the study. Chronic hypertension, assisted reproduction techniques, pre-gestational diabetes, invasive procedure in pregnancy, gestational diabetes and placenta praevia were linearly associated with increasing maternal age whereas hypertensive disorders of pregnancy followed a “U” shaped distribution according to maternal age. Crude rates of preterm birth before 37 weeks followed a “U” shaped curve with a nadir at 5.7% for the group of 30–34 years. In multivariate analysis, the adjusted odds ratio (aOR) of prematurity stratified by age group followed a “U” shaped distribution with an aOR of 1.08 (95%CI; 1.01–1.15) for 20–24 years, and 1.20 (95% CI; 1.06–1.36) for 40 years and older. Confounders found to have the greatest impact were placenta praevia, hypertensive complications, and maternal medical history.

**Conclusion:**

Even after adjustment for confounders, advanced maternal age (40 years and over) was associated with preterm birth. A maternal age of 30–34 years was associated with the lowest risk of prematurity.

## Introduction

During the last decades, a gradual increase of maternal age has been observed worldwide. In the United States, between 1970 and 2006, the proportion of pregnant women aged over 35 years has increased almost eight times [1] and therefore researchers have been interested in outcomes of pregnancy in women of advanced age [2–5]. Pregnancy complications such as placenta praevia, intra-uterine growth restriction or fetal demise, gestational diabetes, hypertensive disorders of pregnancy, and caesarean delivery are well known to be more common in older pregnant women [6–10]. Therefore, guidelines have emerged, both in North America and Europe, for the management of pregnancy in patient with advanced maternal age [11–13].

Preterm birth is the most important factor determining neonatal morbidity and mortality, and has a major impact on it. However, in literature, the association between prematurity and advanced maternal age remains controversial. A study on more than 80,000 women revealed that 36% of the increase in prematurity, between 1990 and 1996 in Canada, was attributable to the change towards increasing maternal age [10]. Various studies have tried to study the specific influence of advanced maternal age after adjustment for hypertensive disorders of pregnancy, maternal medical history or assisted reproduction technologies [9, 14, 15], but the evidence is still conflicting. Thus, as outlined in a systematic review, further research is needed to determine if advanced maternal age is an independent factor for prematurity[16].

The aim of this study was to evaluate the relationship between advanced maternal age and prematurity (both spontaneous and iatrogenic) after controlling for multiple confounders.

## Materials and methods

This is a retrospective cohort study using data obtained from the QUARISMA randomized controlled trial [17]. QUARISMA was a cluster intervention trial designed to assess the effectiveness of a complex intervention with background information and audits targeting a general population in terms of safe and sustainable reduction in the rate of caesarean sections. The intervention targeted physicians and nurses, involved audits of indications for cesarean delivery, provision of feedback to health professionals, and implementation of best practices. It took place in 32 hospitals in the province of Quebec, Canada, from 2008 to 2011 and enabled to collect information on more than 184 000 pregnancies. Trained staff collected information on standardized individual records. In this trial, hospitals were the units of randomization and women were the units of analysis. By designating hospitals as the units of randomization (clusters), the study ensured that all women within a given maternity unit were assigned to the same trial group, thereby reducing the risk of contamination of the intervention effect. Ethics approval was obtained by the Ethics research board of CHU Sainte-Justine (Montreal) under the Study Number 2604, for the completion of the trial, for the creation of the database and for the present study.

Inclusion criteria were those of the QUARISMA trial: birth at or after 24 gestational weeks of a fetus weighing >500 grams; and maternal age >20 years. Non-inclusion criteria were multiple pregnancies, fetal malformations and intra-uterine fetal demise.

Five maternal age categories were defined: 20–24, 25–29, 30–34, 35–39 and 40 years and older. Groups of age were compared based on maternal history: past drug use, nulliparity, and medical history including chronic hypertension, diabetes mellitus, renal and cardiac disease, thrombophilia, systemic erythematous lupus and inflammatory bowel disease. Characteristics of the current pregnancy were also studied: drug use, smoking, use of assisted reproductive technologies, and occurrence of an invasive procedure (chorionic villus sampling or amniocentesis). Additionally, groups of age were also compared according to maternal and obstetrical complications: hypertensive complications (gestational hypertension, pre-eclampsia and eclampsia), gestational diabetes and placenta praevia. All comparisons used chi-square test.

The odds ratios for preterm birth (<37 weeks) and very preterm birth (< 32 weeks) were calculated for different age groups before and after adjustment by multivariate logistic regression for known risk factors, maternal characteristics and gestational complications. For these analyses, the reference group corresponded to the group with the lowest rate of prematurity. As our analyses did not focus on the intervention of the primary trial (caesarean section) and since this intervention did not condition the relationship between the explanatory variables and the outcome studied in our paper; we did not performed mixed model analyses accounting for cluster (hospitals).

Preterm birth <37 weeks was divided into spontaneous and iatrogenic preterm birth. For both conditions, risk factors were studied using multivariate logistic analyses after adjustment on covariates. Iatrogenic delivery was defined as performance of a cesarean delivery before onset of labor or induction of labor using cervical ripening or oxytocin.

Results were considered significant when p<0.05. All statistical analyses were performed with the use of SAS software, version 9.3 (SAS Institute)

## Results

**Fig 1:**
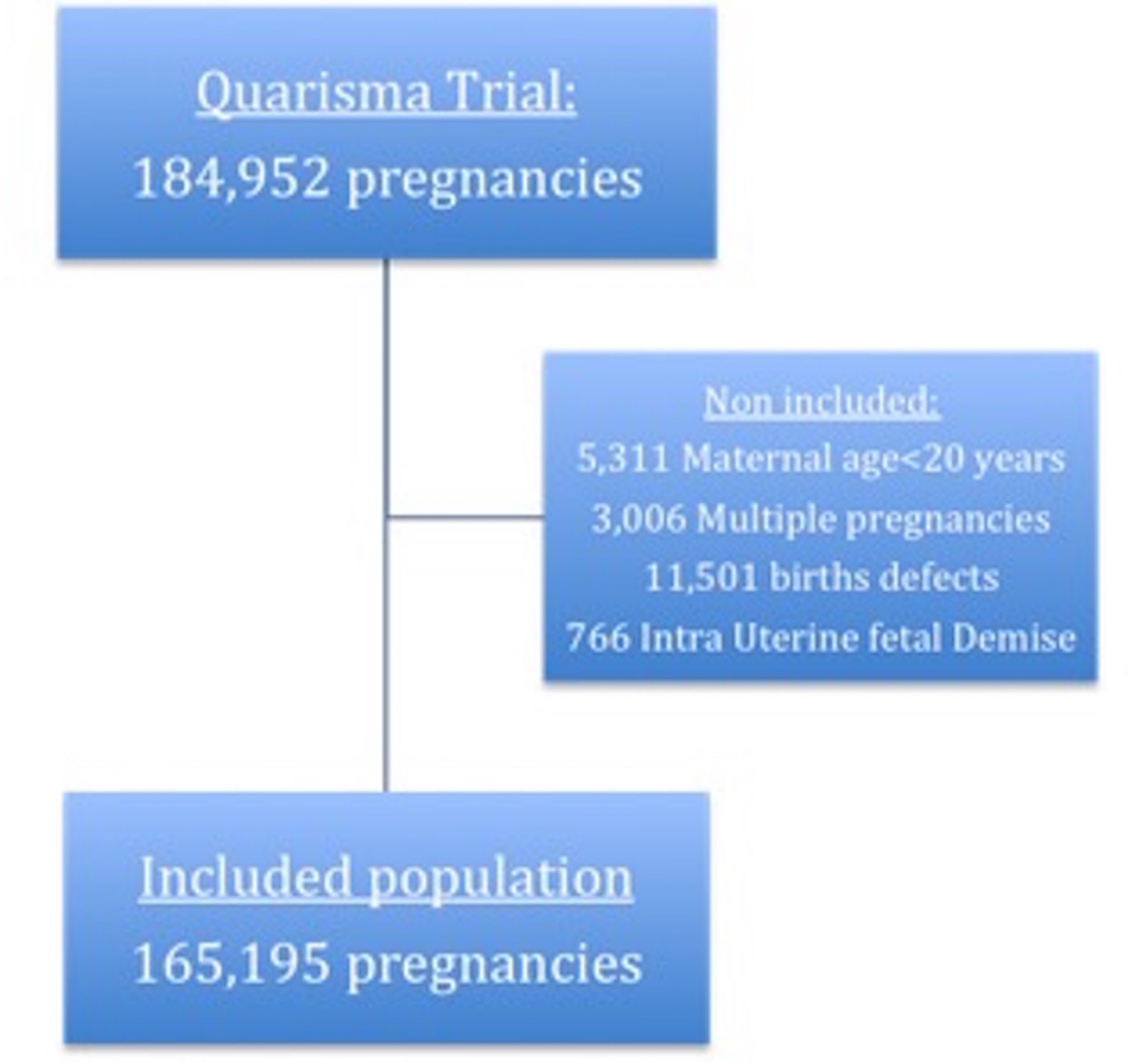
Flow chart.

QUARISMA trial reported the outcome of 184,952 deliveries. After exclusions, a total of 165,195 births were finally included in the study and distributed as follows: 24 650 aged 20–24 years; 59 124 aged 25–29 years; 55 867 aged 30–34 years; 21 416 aged 35–39 years; 4138 aged 40 years or more (Fig 1).

Comparison of excluded (19,757) and included (165,195) births did not show any discrepancy regarding maternal distribution of age or maternal characteristics. Risk factors for prematurity by age category are presented in Table 1. Compared to the 30–34 years old group, the rate of chronic hypertension almost tripled in the >40 years group (4.1% versus 1.4%) and the rate of gestational diabetes more than doubled (19.4% versus 8.7%). The rates of pre-existing diabetes, assisted reproductive technologies, invasive procedure, placenta praevia and obesity also increased with maternal age. The prevalence of hypertensive disorders were higher among extreme of ages: the rates of gestational hypertension were lowest in patients aged 30 to 34 years, and the rates of preeclampsia were lowest in patients aged 25 to 34 years.

**Table 1:** Prevalence of maternal and obstetrical risk factors of prematurity by age group.

	Overall		20–24 years		25–29 years		30–34 years		35–39 years		40 years and over		p value*
	n	%	n	%	n	%	n	%	n	%	n	%	
	165195		24650		59124		55867		21416		4138		
**Maternal history**													
Past drug use, n (%)	3292	2,0%	1107	4,5%	1199	2,0%	715	1,3%	231	1,1%	40	1,0%	< .001
Primiparity, n (%)	71145	43,1%	16095	65,3%	29775	50,4%	18802	33,7%	5450	25,4%	1023	24,7%	< .001
**Past medical history**													
Chronic hypertension, n (%)	2349	1,4%	211	0,9%	683	1,2%	792	1,4%	493	2,3%	170	4,1%	< .001
Diabetes mellitus without insulin, n (%)	555	0,3%	32	0,1%	123	0,2%	192	0,3%	167	0,8%	41	1,0%	< .001
Diabetes mellitus with insulin, n (%)	653	0,4%	60	0,2%	230	0,4%	221	0,4%	118	0,6%	24	0,6%	< .001
Renal disease, n (%)	2217	1,3%	338	1,4%	752	1,3%	736	1,3%	340	1,6%	51	1,2%	.013
Cardiac disease, n (%)	2939	1,8%	419	1,7%	1057	1,8%	950	1,7%	443	2,1%	70	1,7%	.010
Thrombophilia, n (%)	2009	1,2%	201	0,8%	652	1,1%	749	1,3%	323	1,5%	84	2,0%	<0.01
Systemic erythematous lupus, n (%)	111	0,1%	4	0,0%	33	0,1%	49	0,1%	19	0,1%	6	0,1%	< .001
Crohn disease, n (%)	927	0,6%	88	0,4%	328	0,6%	355	0,6%	134	0,6%	22	0,5%	< .001
**Characteristics of current pregnancy**													
Drug use, n (%)	4290	2,6%	1583	6,4%	1524	2,6%	864	1,5%	258	1,2%	61	1,5%	< .001
Smoking, n (%)	23820	14,4%	6962	28,2%	8675	14,7%	5811	10,4%	1958	9,1%	414	10,0%	< .001
Assisted reproductive technologies, n (%)	2073	1,3%	51	0,2%	452	0,8%	846	1,5%	546	2,5%	178	4,3%	< .001
Invasive procedure, n (%)	6157	3,7%	213	0,9%	776	1,3%	1210	2,2%	2762	12,9%	1196	28,9%	< .001
**Maternal and obstetrical complications**													
Hypertensive complications, n (%)	11496	7,0%	1854	7,5%	4075	6,9%	3516	6,3%	1610	7,5%	441	10,7%	< .001
At least one hypertensive complication													
Gestational hypertension without adverse criteria	5415	3,3%	852	3,5%	1993	3,4%	1675	3,0%	714	3,3%	181	4,4%	< .001
Gestational hypertension with adverse criteria	1360	0,8%	206	0,8%	512	0,9%	415	0,7%	170	0,8%	57	1,4%	< .001
Pre-eclampsia without adverse criteria	2536	1,5%	440	1,8%	847	1,4%	761	1,4%	387	1,8%	101	2,4%	< .001
Pre-eclampsia with adverse criteria	2108	1,3%	342	1,4%	705	1,2%	641	1,1%	321	1,5%	99	2,4%	< .001
Eclampsia	77	0,0%	14	0,1%	18	0,0%	24	0,0%	18	0,1%	3	0,1%	.026
Gestational diabetes, n (%)	13335	8,1%	1152	4,7%	3573	6,0%	4848	8,7%	2960	13,8%	802	19,4%	< .001
Placenta preavia, n (%)	1207	0,7%	99	0,4%	328	0,6%	458	0,8%	266	1,2%	56	1,4%	< .001
Obesity (n = 118 347), n (%)	20954	17,7%	3204	17,8%	7417	16,9%	6896	17,4%	2887	20,2%	550	21,4%	< .001

(pvalue* correspond to overall comparison).

**Fig 2:**
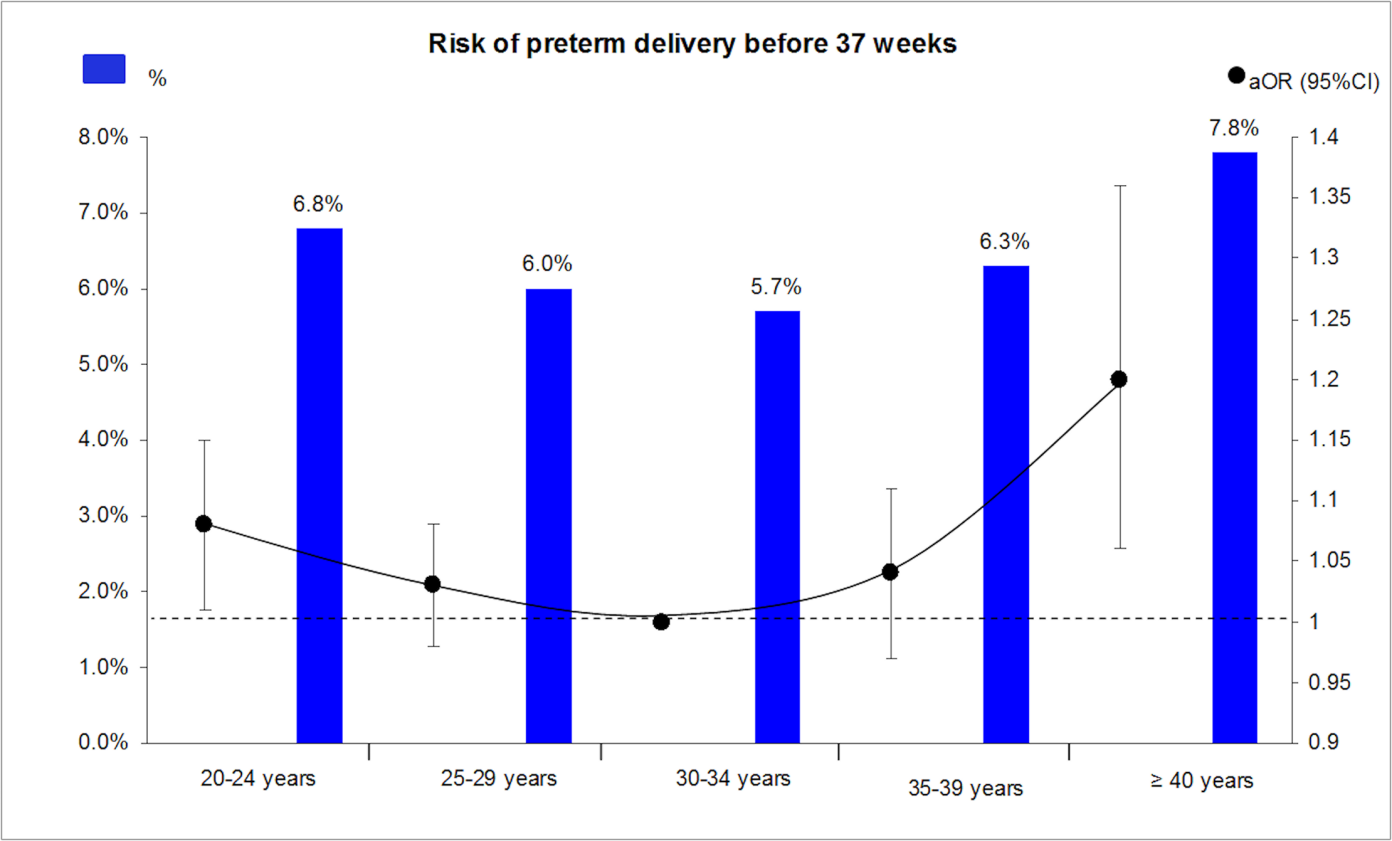
Risks of all preterm delivery (spontaneous and iatrogenic) before 37 weeks and adjusted odds ratio according to maternal age categories.

Rates of preterm birth <37 weeks and very preterm birth <32 weeks were lowest in the 30–34 years old group (5.7% and 0.6% respectively) and highest in women over 40 years (7.8% and 1.0% respectively) (Table 2 and Fig 2). Crude and adjusted odds ratios (ORs, aORs) for preterm birth, very preterm birth, iatrogenic and spontaneous preterm delivery before 37 weeks, are presented in Table 2. For mothers younger than 24 years and older than 35 years, preterm birth was significantly more frequent compared to the reference group (30–34 years). There was a trend towards increased risk in women aged 25–29 years. ORs for preterm birth, extreme preterm birth, and spontaneous preterm birth in the group of 40 years or more were respectively 1.39 (95% CI 1.24–1.57), 1.68 (95% CI 1.21–1.31) and 1.20 (1.04–1.39). Iatrogenic prematurity was almost twice as common in this group (OR 1.91 (95% CI 1.56–2.34)).

**Table 2:** Risk of preterm birth and very preterm birth according to maternal age.

	Overall	20–24 years	25–29 years	30–34 years	35–39 years	40 years and over
N	165195	24650	59124	55867	21416	4138
Gestational age <32 weeks						
N (%)	1120 (0,68%)	206 (0,84%)	370 (0,63%)	340 (0,61%)	162 (0,76%)	42 (1,01%)
Crude OR (95% CI)		1.38 (1.16–1.64)	1.03 (0.89–1.19)	1.00	1.25 (1.03–1.50)	1.68 (1.21–2.31)
Adjusted OR* (95% CI)		1.16 (0.97–1.39)	0.97 (0.83–1.13)	1.00	1.14 (0.94–1.38)	1.33 (0.94–1.86)
Gestational age <37 weeks						
N (%)	10085 (6,1%)	1664 (6,75%)	3554 (6,01%)	3202 (5,73%)	1342 (6,27%)	323 (7,81%)
Crude OR (95% CI)		1.19 (1.12–1.27)	1.05 (1.00–1.10)	1.00	1.10 (1.03–1.17)	1.39 (1.24–1.57)
Adjusted OR* (95% CI)		1.08 (1.01–1.15)	1.03 (0.98–1.08)	1.00	1.04 (0.97–1.11)	1.20 (1.06–1.36)
Spontaneous preterm birth <37 weeks	7683 (4,7%)	1308 (5,3%)	2797 (4,7%)	2423 (4,3%)	941 (4,4%)	214 (5,2%)
Crude OR (95% CI)		1.23 (1.15–1.32)	1.10 (1.04–1.16)	1.00	1.01 (0.94–1.10)	1.20 (1.04–1.39)
Adjusted OR* (95% CI)		1.09 (1.02–1.18)	1.06 (1.00–1.12)	1.00	0.99 (0.92–1.08)	1.14 (0.98–1.31)
Iatrogenic preterm birth < 37 weeks	2402 (1,5%)	356 (1,4%)	757 (1,3%)	779 (1,4%)	401 (1,9%)	109 (2,6%)
Crude OR (95% CI)		1.04 (0.91–1.18)	0.92 (0.83–1.01)	1.00	1.35 (1.20–1.52)	1.91 (1.56–2.34)
Adjusted OR* (95% CI)		1.02 (0.89–1.17)	0.92 (0.83–1.02)	1.00	1.15 (1.01–1.31)	1.31 (1.05–1.64)

*Adjustment was performed for primiparity, past medical history (chronic hypertension, pre-gestational diabetes, renal disease, cardiac disease, thrombophilia, systemic erythematous lupus and crohn disease), smoking status, drug use (past or current), use of assisted reproductive technologies, occurrence of an invasive procedure, hypertensive complications, gestational diabetes and placenta praevia.

After adjustment for potential confounders, advanced maternal age (40 years and over), compared to the reference group (30–34 years), was associated with preterm birth <37 weeks and iatrogenic preterm birth (aOR 1.20 (95% CI 1.06–1.36) and aOR 1.31 (95% CI 1.05–1.64) respectively). Age 35–39 years was also associated with iatrogenic prematurity (aOR 1.15 (1.01–1.31)). Younger women (20–24 years) had an increased risk of preterm birth (aOR 1.08 (95% CI 1.01–1.15) and spontaneous preterm birth (aOR 1.09 (95% CI 1.02–1.18). Detailed results of the multivariate analysis are presented in Table 3. Placenta praevia and hypertensive disorders were associated with the highest risk for preterm birth <37 weeks, due to the increase risk in iatrogenic preterm birth<37 weeks.

**Table 3:** Value of adjusted ORs to predict preterm delivery before 32 weeks and 37 weeks.

	Delivery < 32 weeks	Delivery < 37 weeks
	aOR (95% CI)	aOR (95% CI)
Maternal history		
Past drug use, n (%)	1.35 (0.96–1.91)	1.09 (0.95–1.25)
Nulliparity, n (%)	1.57 (1.39–1.78)	1.20 (1.15–1.26)
Past medical history*	2.44 (2.06–2.88)	1.82 (1.70–1.94)
Characteristics of current pregnancy		
Smoking, n (%)	1.22 (1.03–1.44)	1.35 (1.28–1.43)
Drug use, n (%)	1.86 (1.38–2.51)	1.50 (1.34–1.69)
Assisted reproductive technologies, n (%)	1.58 (1.06–2.33)	1.27 (1.08–1.49)
Fetal invasive procedure, n (%)	1.67 (1.29–2.16)	1.18 (1.07–1.31)
Complications of pregnancy		
Hypertensive disorders, n (%)	1.66 (1.26–2.19)	2.07 (1.88–2.29)
Gestational diabetes, n (%)	1.11 (0.91–1.36)	1.36 (1.28–1.46)
Placenta preavia, n (%)	7.06 (5.31–9.39)	7.05 (6.21–7.99)

* Chronic hypertension, pre-gestational diabetes, renal disease, cardiac disease, thrombophilia, systemic erythematous lupus and Crohn disease.

## Discussion

We found that advanced maternal age (40 years and over) was associated with an increased risk of preterm birth even after adjustment for confounders. The lowest risk of prematurity was found in mothers aged 30–34 years. Preterm birth was mainly spontaneous in younger women (20–24 years) whereas it was more frequently of iatrogenic origin in women over 40.

Our results are in accordance with those of two recently published cohort studies. Lawlor et al, in a population of Danish women, found a U shaped relationship between maternal age and risk of preterm birth, with the lowest risk age at 24–30 years [18]. A more recent nationwide register-based cohort study in Finland found that the threshold-ages for preterm birth was 28 years (OR 1.10, 1.02–1.19) [5]. However the authors used different inclusion criteria and they did not stratify their results according to the onset of preterm birth (spontaneous or iatrogenic)

Confounders identified in our study are known risk factors for prematurity. Placenta praevia, gestational diabetes, medical history, use of assisted reproduction technologies and occurrence of an invasive procedure were all more common in aged mothers. On the other hand, nulliparity, past drug use and smoking were more prevalent in younger mothers. Furthermore, the prevalence of hypertensive disorders was lowest in middle-aged groups. This distribution of risks factors probably accounts for the “U” shaped distribution of preterm birth risk among age groups. Past research has already shown that younger mothers tend to have higher prematurity rates, but the persistence of this effect until 30 years old has rarely been identified [19]. In contrast, some studies have found a higher risk of preterm birth risk among women of the age group 30–34 years [3, 5, 20–22]. This difference could be explained by variations in socio-demographic or clinical risk factors across different studies.

A common hypothesis is that the increased risk of preterm birth among aged mothers is largely explained by early labor induction for medical conditions. However, our analysis of iatrogenic versus spontaneous prematurity rates among aged mothers does not confirm this hypothesis. Khalil et al. found opposite results in a recent cohort study [23]. This discrepancy could be due to a different definition of iatrogenic preterm birth. In our study, the variable “iatrogenic preterm birth” was generated using a combination of other variables describing the method of induction of labor. Such data are exposed to classification bias by data abstractors, and some preterm births could have been misclassified. For example, preterm births by caesarean section secondary to preterm premature rupture of membranes could have been misclassified as iatrogenic because of an “elective caesarean section” at 34 or 36 weeks. Iatrogenic preterm births could have been misclassified as spontaneous if oxytocin induction was confounded with oxytocin augmentation. Nevertheless, in light of our results, we cannot rule out that advanced maternal age is independently associated with spontaneous prematurity, as McIntyre et al. concluded in a population based cohort study [20]. Regarding younger women (20–24 years), we confirmed that preterm birth was mainly spontaneous rather than iatrogenic. As most women delay their first pregnancy at a later age, women who still become pregnant at a young age mainly correspond to low socioeconomic status women with higher risk of medical complication of pregnancy. Even if this study controlled a large number of variables, we could not adjust on educational level or social insurance as this was not reported in the initial study.

The principal strength of this study is the size of the cohort with more than 165 000 patients studied. Furthermore, the sampling represents a broad spectrum of patients, including patients from rural and urban communities across a Canadian province. This prospective cohort nested in a large and well-designed randomized controlled trial allowed controlling for a large number of variables, with a standardized data collection and a strict quality control. Hence, the confounding effect of data such as the use of assisted reproductive technologies and occurrence of an invasive procedure has rarely been studied. Yet these factors are important, with aORs for extreme prematurity of 1.58 (95% IC 1.06–2.33) and 1.67 (95% IC 1.29–2.16).

This study has some limitations. Some potential confounders could not be studied. BMI information was missing in 28% of patients, therefore, it was not used in multivariate analysis. In the population studied, obesity was more common in advanced maternal age mothers. Previous research has shown that excess weight is associated with overall prematurity before 32 weeks and induced prematurity before 37 weeks [24]. Thus, controlling for BMI could have yielded different results. Moreover, socio-economic data were not available in the database we used. However, a previous study has shown that in older mothers, the association between maternal age and preterm birth was not explained by a confounding effect of socio-economic status[18]. Another limitation of the study is that we could not adjust for history of preterm delivery. Even though this variable was reported in the database, it was excluded from the final analysis, due to misclassification and lack of reliability after quality control. However, it is unlikely that previous preterm delivery would be more frequent in older women, thus reducing the risk of a confounding effect of previous preterm delivery.

## Conclusion

In conclusion, this study based on a large birth cohort was able to demonstrate that even after adjustment for many potential confounders known to be associated with preterm birth, advanced maternal age was independently associated with preterm delivery. Women of 30–34 years had the lowest risk of preterm delivery.
